# Protective efficiacy of taurine against pulmonary edema progression: experimental study

**DOI:** 10.1186/1749-8090-3-57

**Published:** 2008-10-28

**Authors:** Orhan Yucel, Zeki Ilker Kunak, Enis Macit, Armagan Gunal, Alper Gozubuyuk, Husamettin Gul, Onur Genc

**Affiliations:** 1 Department of Thoracic Surgery, Gulhane Military Medical Academy, Ankara, Turkey; 2 Department of Medical CBRN, Gulhane Military Medical Academy, Ankara, Turkey; 3 Department of Analytical Toxicology, Gulhane Military Medical Academy, Ankara, Turkey; 4 Department of Pathology, Gulhane Military Medical Academy, Ankara, Turkey

## Abstract

Re-expansion pulmonary edema (RPE) is an acute, rare and potentially lethal complication [1,2]. Its beginning is sudden and dramatic. The mechanism is not yet fully understood [1]. Some authors suggest that it may occur after rapid re-inflation of a collapsed lung [1]. It was reported by other authors that it may relate to surfactant depletion or may result from hypoxic capillary damage, leading to increased capillary permeability [1,3]. In RPE, unilateral lung injury is initiated by cytotoxic oxygen metabolites and temporally associated with an influx of polymorphonuclear neutrophils [1]. These toxic oxygen products are the results of re-oxygenation of a collapsed lung. Treatment of re-expansion pulmonary edema is basically preventive [4].

## Introduction

Re-expansion pulmonary edema (RPE) is an acute, rare and potentially lethal complication [1,2]. Its beginning is sudden and dramatic. The mechanism is not yet fully understood [1]. Some authors suggest that it may occur after rapid re-inflation of a collapsed lung [1]. It was reported by other authors that it may relate to surfactant depletion or may result from hypoxic capillary damage, leading to increased capillary permeability [1,3]. In RPE, unilateral lung injury is initiated by cytotoxic oxygen metabolites and temporally associated with an influx of polymorphonuclear neutrophils [1]. These toxic oxygen products are the results of re-oxygenation of a collapsed lung. Treatment of re-expansion pulmonary edema is basically preventive [4].

The RPE is a disease process that is characterized by diffuse inflammation in the lung parenchyma and resultant permeability edema [2]. The involvement of inflammatory mediators in RPE has been the subject of intense investigation, and oxidant-mediated tissue injury is likely to be important in the pathogenesis of RPE [2]. In response to various inflammatory stimuli, lung endothelial cells, alveolar cells, and airway epithelial cells, as well as alveolar macrophages, produce reactive oxygen species (ROS) [2,5]. Free oxygen radicals increase after pulmonary re-expansion and, it can enhance the production of these toxic species [2,5]. As the antioxidant defense system, various enzymes and low-molecular weight ROS scavengers are present in the lung tissue and epithelial lining fluid [2,5].

Taurine, 2-amino ethane sulfonic acid, is a normal constituent of the human diet and is a ubiquitous powerful antioxidant [6]. It prevents tissue injury mainly through antioxidation [6]. Taurine was revealed to be beneficial in preventing experimental lead-induced oxidative damage, diabetic neuropathy, CCl_4_-induced oxidative stress, caeurelein-induced acute pancreatitis, and early changes in experimental diabetic kidney through antioxidant mechanisms [6-8]. In bleomycine-induced lung injury and glomerular basal membrane damage caused by stimulated neutrophils which may be activated by oxidative stress and preventive effect of taurine were reported [7]. Previous studies have shown that taurine exhibits a protective effect against cellular-stress induced oxidation [8,14]. Indeed, taurine behaves as a free radical scavenger in various cells and tissues [9-11,14]. The protective effects of taurine against cytotoxicity and oxidative stress have been observed in cells and tissues, both in vivo and in vitro [8,9,11,14]. Since we think that cytotoxic oxygen metabolites are important in the mechanism of RPE, we aimed to investigate the possible beneficial protective effects of taurine in RPE in rats.

## Materials and methods

The study was performed in Gulhane Military Medical Academy (GMMA) Animal Research Laboratory. GMMA Ethics committee's permission was obtained before the study.

### Specifications of Laboratory Animals

In this study, 21 adult Spraque-Dawley rats (weighing: 150 +/- 20 g) were used. Prior to the initiation of the experiment, all animals were provided access to the same feed (2650 kkal/kg metabolic energy, 22% protein, 8% cellulose, and fat 8% and water in the same environment, 20–22°C) for a period of 7 days.

### Experimental design

21 adult rats were subdivided into three groups by the simple random sampling method. The first group was the control group (CG), the second group was RPE group (RPEG) and the third group was RPE plus taurine group (TG). All animals were subject to the same experimental protocol. RPE or PA wasn't performed in CG. The RPE was performed in RPEG and TG. In addition, TG was given taurine containing diet.

### Antioxidant Agent Application

TG was given taurine (200 mg/kg rat body weight) containing solution orally by gavage.

The RPE procedure is explained below in details.

### The RPE Procedure

Rats were anesthetized with intraperitoneal Ketamine Hydrocloride (Ketamine hydrochloride solution in %5, Parke – Davis license Eczacıbaşı Medical Industry, Ýstanbul) 90 mg/kg and Xylazine (Xylazine solution in %2, by Parke – Davis license Eczacıbaşı Medical Industry, Ýstanbul, 10 mg/kg). 200 mg taurine was diluted with 3 ml of 0.9% NaCl solution. In TG, taurine administration was started 8 h before pneumothorax application and continued for 72 hours by gavage.

All rats' right chest walls were shaved. In RPEG and TG, pneumothorax was induced by injecting about 4 ml of air into the thorax via percutaneous route with a 22 gauge cannula which was placed in the right hemithorax.

**Figure 1:**
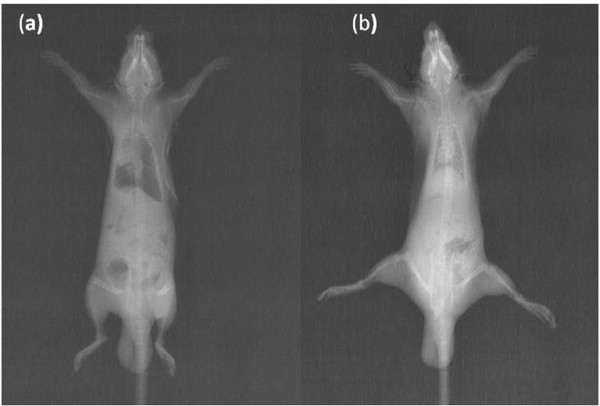
The pneumothorax and reexpancion were confirmed with control X-rays in all groups, (a) we ensured the pneumothorax by X-ray graphs (b) chest X-ray confirmed right re-expansion.

The adequacy of the pneumothorax was confirmed with control X-rays in all groups (figure 1a). Thereafter, the animals were allowed to survive for an additional 72 h. Analgesia was obtained by using buterfenol (0,5 mg/kg sc). After 72 hours, in all rats, pneumothorax was immediately recovered by aspiration with a 22 gauge cannula. The adequacy of the reexpancion was confirmed with control X-rays (figure 1b) at onset and after 72 hours by sternotomy in all groups. All rats were sacrificed by giving lethal dose of xylazine and ketamine 2 hours after reexpancion of pulmonary. Their chest was opened by median sternotomy. Then, the lungs were removed immediately for histopathological and histochemical evaluation. Histopathological samples of lungs were fixed in 10% buffered formaldehyde. Histochemical samples were kept in liquid nitrogen for oxidative stress status analysis.

### Tissue preparation for histopathological evaluation

After fixing in 10% buffered formaldehyde the lung samples were embedded in paraffin blocks. 4 μm sections were sliced from paraffin blocks and stained with hematoxylin-eosin (HE). Pulmonary edema was evaluated by blinded two pathologists for the research. The frozen tissues were homogenized in phosphate buffer (pH 7.4) by means of a homogenizator (Heidolph Diax 900; Heidolph Elektro GmbH, Kelhaim, Germany). The supernatant was divided into 2–3 parts, put in separate tubes, and stored at -70°C.

### Tissue preparation for oxidative stress status

We used the same methods that Ucar et al had previously used in determining oxidative stress and antioxidant enzymes [12]. Since we had to express the data we had as per tissue protein, we assayed the protein content of the lung. With the thiobarbituric acid (TBA) reaction lipid peroxidation was measured. In this method, during the reaction of thiobarbituric acid (TBA) with malondialdehyde (MDA) at 535 nm a color was produced and this was used to obtain a spectrophotometric measurement. We reported the final estimated MDA levels in mmol/g-protein in lung tissue. We determined the cupper/zinc-superoxide dismutase (Cu/Zn-SOD) activity with the nitroblue tetrazolium (NBT) method. In this method NBT is reduced to formazan by the superoxide radical (O^-2^) and this compound has a strong absorbance at 560 nm. In this study, one unit (U) of Cu/Zn-SOD means the amount of protein that inhibits the rate of NBT reduction by 50%. U/g-protein was used to express the calculated enzyme activity. Glutathione peroxidase (GPx) activity was assessed with the method where GPx is coupled via the oxidation of NADPH by glutathione reductase. With a spectrophotometer, the oxidation of NADPH had been followed up at 37°C for 5 minutes, and the absorbance at 340 nm was recorded. Since mmol of NADPH oxidized per minute formed a line in the graphic, the slope of the line meant us the activity. U/g-protein is used for GPx activity.

### Statistical analysis

In the statistical procedure of our study we used Duncan test besides one-way analysis of variance, and all these statistical analyses had been performed by statistical software package SPSS 11.0 for Windows. We used Kruskal-Wallis and Mann-Whitney U tests to statistically analyse the each group mutually. We stated the histopathological and histochemical results as the median (min-max) where p < 0.05 was found statistically significant.

## Results

### Histopathological evaluation

The final results of the performed histophatological examination were; normal pulmonary parenchyma, fluid extravasations, fluid extravasations with fluid in the alveoli and pulmonary edema. Where these findings represent a pulmonary edema, the extravasation of fluid is the most common finding in histophatological examination. The results of this histopathological examination are presented in table 1.

**Table 1:** Histopathology results of the groups.

	**CG**	**RPEG**	**TG**
n	10	10	10
Normal Pulmonary Parenchyma	10	0	4^**β, μ**^
Fluid Extravasations	0	2 ^**α**^	4^**μ**^
Fluid Extravasations with Fluid In The Alveoli	0	6^**α**^	2^**β**^
Real reexpancion Pulmonary Edema	0	2^**α**^	0^**β**^

CG; Control Group, RPEG; Re-expansion Pulmonary Edema Group, TG; Taurine Treatment Group. α:   p < 0,05, RPEG compared with CG; β: p < 0,05, TG compared with RPEG, μ: p < 0,05, TG compared with CG.

**Figure 2:**
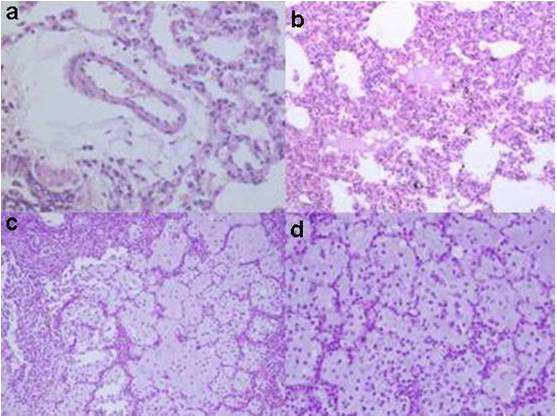
(a) fluid extravasation in the perivascular areas, (HEx200), (b) esinophilic fluid accumulation in some of the alveolar spaces (HEx100), (c) severe pulmonary edema with alveolar damage and scattered typical acute inflamatory cells in RPE (HEx400), (d) severe pulmonary edema with alveolar damage and scattered typical acute inflamatory cells in RPE (HEx200).

The most noticeable finding is real reexpantion pulmonary edema and it occurred in only two animals in the RPEG group. The severity of the pulmonary edema was significantly reduced in the Taurine administrated group. Real reexpantion pulmonary edema's characterization includes severe pulmonary edema with alveolar damage and acute inflammation. Various severity levels of reexpantion pulmonary edema are shown at the figure 2. In RPEG group animals, accumulations of alveolar macrophages with sparse neutrophilic infiltrate at the alveolar and interstitial edema regions are indicated.

### MDA levels, GPx and SOD activities in tissue

Oxidative stress status analysis included MDA level, SOD and GPx activity. RPE caused increased MDA levels, and decreased GPx and SOD activity significantly in lung tissue (P < 0.05) (Table 2). Taurine treatment decreased MDA levels and increased SOD and GPx activities compared with RPEG (P < 0.05). MDA in RPEG was much higher than that in TG (P < 0.05). In addition, SOD and GPx activities in TG were approximately as same as activities of those in Group CG (P > 0.05).

**Table 2:** Oxidative stress related parameters of the groups.

**Groups**	**n**	**MDA (nmol/g)** **Mean ± Std. Deviation**	**SOD (U/g)** **Mean ± Std. Deviation**	**GPx** **Mean ± Std. Deviation**
**CG**	10	1,45 ± 0,21	654,48 ± 98,48	24,94 ± 1,94
**RPEG**	10	1,78 ± 0,12 α	585,32 ± 97,14 α	20,44 ± 1,53 α
**TG**	10	1,63 ± 0,32 β	655,32 ± 112,11 β, μ	23,85 ± 2,46 β

Contrtol group (CG), RPEG Reexpancion Pulmonary Group and Taurine groups (TG) lung tissues. MDA; Malondialdehyde, SOD; Superoxide Dismutase. α: p < 0,001, RPEG compared with CG; β: p < 0,05, TG compared with RPEG, μ: p < 0,05, TG compared with CG.

## Discussion

There are some studies about reinflation of a collapsed lung can lead to pulmonary edema of the reexpanded lung [5]. RPE has a mortality rate changing from 0% to 20% [3,13]. Some of the patients' conditions who had severe RPE further deteriorated leading to bradycardia, hypotension, cardiopulmonary arrest and death [4].

There are some studies about causes of RPE [2]. It is emphasized that the most important reason of RPE is the prolonged collapse of a lung for 72 hours before reexpancion [2]. In the light of these knowledge, in our study, we kept the lungs collapsed for 72 hours to develop RPE. Re-expansion of a collapsed lung increases the microvascular permeability and causes reexpansion pulmonary edema [5,14]. Neutrophils and their products have been implicated in the development of this phenomenon [14]. There is a large body of evidence that reactive oxygen species produced during RPE play a key role in lung damage and endothelial dysfunction [15]. It was suggested that antioxidants might play a role in the treatment of RPE. Taurine is known to be a potent antioxidant and a membrane-stabilizing agent. Therefore; we investigated whether taurine has a possible beneficial effect in RPE in rats.

Previous studies demonstrated that administration of taurine, vitamin C, and vitamin E partially protected from oxidative damage and while all substances had antioxidant effects, only taurine showed morphological protection in surviving cells [16]. Other studies have demonstrated that addition of taurine to St. Thomas' cardioplegic solution, improved cardiac function recovery for prolonged hypothermic rat heart preservation by suppressing DNA oxidative stress and cell apoptosis [17]. Besides this, another study have emphasized that treatment with taurine reduces iron-mediated myocardial oxidative stress, preserves cardiovascular function, and improves survival in iron-overloaded mice. Oudit et al revealed the role of taurine in protecting reduced glutathione levels that provides an important mechanism by which oxidative stress-induced myocardial damage can be curtailed [18]. All these studies made us choose taurine to use as a potent antioxidant against RPE.

Sivrikoz et al reported that lung is the largest reservoir for monocytes, macrophages and PNLs. In their study, lymphocyte (++) dominance was found semi-quantitatively at the end of the pnomothorax. Together with reexpancion, lymphocyte levels were increased (+++) and PNLs started to remigrate to the area. They empahsized that the reperfusion achieved during reexpancion increases the production of free radicals by activating the inflammatory cells in addition to increasing the oxygen supply to the tissue [1].

Saito et al revealed that they therefore believe ROS produced by xanthine oxidase in endothelial and alveolar type II cells, plays a major role in the pathogenesis of RPE [19].

Doerschunk et al demonstrated that histological examination of the injured right lungs in control animals has focal areas of edema; hemorrhage in both the alveolar spaces and the interstitium. There was lymphatic dilatation and edema within the bronchovascular bundle. They founded that alveolar macrophages containing cytoplasmic granules were more in number than PML which are sparse in the injured lung [5].

In our study, the most common histophatological findings in all groups were fluid extravasations with fluid in the alveoli. The other important finding was a real pulmonary edema. It is very important to improve ARDS [15]. Because of ineffective treatment, developed ARDS is very mortal complication. In RPEG group animals, accumulations of alveolar macrophages with sparse neutrophilic infiltrate at the alveolar and interstitial edema regions are indicated.

There is a large body of evidence that reactive oxygen species (ROS) produced during RPE play a crucial role in lung damage and endothelial dysfunction [1]. Therefore, oxidative stress parameters like MDA level, SOD and GPx activities in rats were determined following taurine treatment. RPE caused significantly increased MDA levels, and decreased GPx and SOD activity in lung tissue in our study. On the other hand, taurine treatment increased SOD activity and decreased MDA levels. Taurine was found to be effective in reducing malondialdehyde levels also.

Treatment of RPE is supportive [4]. New drugs has been studied to avoid or to treat reexpansion edema [4]. Ibuprofen and misoprostol are routinely used for their anti inflammatory and cell protecting effects in the clinical practice [20]. The use of monoclonal antibodies prevented neutrophilin filtration and acute pulmonary lesion induced by the reperfusion ischemia process [21]. It is suggested that the use of monoclonal antibodies in high risk patients could be an effective in protective therapy [21]. In our study, because it is a strong antioxidant agent, we used taurine for preventing and treatment of RPE. Briefly, the purpose of using taurine in the diagnostic hypothesis during the initial symptom phases is protecting or reducing the risk of developing of lesions that lead RPE. We demonstrated the beneficial effect of taurinee in preventing the pulmonary edema in hystopathological examination.

Our study presented that taurine administration has useful effects against the lung injury after reexpancion. The mechanisms of the protective effects of taurine may be related to the decrease of MDA level and the increase of SOD and GPx activity. This experimental study shows that supplementation of taurine is safe and seems to have positive influence on the subjects with RPE. Although we indicated that taurine might be effective in preventing, protecting and in the treatment of RPE, further studies are needed to be done about this subject. For this reason, we are planning some consecutive studies related with the topics: 1) Proper treatment dosage 2) Suitable administration way of taurine, and 3) Most appropriate treatment interval time.

## Competing interests

The authors declare that they have no competing interests.

## Authors' contributions

OY was involved with study design, performed the data analysis and all the operations. HG was designed the study and performed data analysis. AG did the background literature search. ZIK collected and entered the data. The lung samples were evaluated by AG. EM prepared the images for the manuscript. OG was designed the study and has given final approval of the version to be published. All authors have read and approved the manuscript.
